# Colorectal Endoscopic Submucosal Dissection and Acute Kidney Injury: A Rare but Clinically Relevant Adverse Event, a Cohort Study of 1537 Patients

**DOI:** 10.1055/a-2914-8234

**Published:** 2026-09-23

**Authors:** Eugenio Baglione, Veronique Van der Voort, Zhour El Ouafi, Jérémie Albouys, Romain Legros, Sara Battistella, Hugo Lepetit, Clément Juglard, Remi Collin, Pauline Champy, Bertrand Landreau, Youssef Mlaiki, Nora Boutarbouch, Clement Danthu, Constance Grunewald, Sophie Geyl, Paul Carrier, Mathieu Pioche, Fatouma Toure, Jérémie Jacques

**Affiliations:** 1 Hepatogastroenterology Department 36715 CHU LimogesLimogesAquitaine-Limousin-Poitou-CharentesFrance; 2 Gastroenterology UnitDepartment of Surgery, Oncology and GastroenterologyPadua UniversityPaduaItaly; 3 Nephrology, Dialysis and Transplantation Department, and UMR-CNRS 7276 INSERM U1262 CRIBL 36715 CHU LimogesLimogesAquitaine-Limousin-Poitou-CharentesFrance; 4 IDIBAPS, CIBEREHD 16493 Hospital Clinic de BarcelonaBarcelonaSpain; 5 Anaesthesiology Department 36715 CHU LimogesLimogesAquitaine-Limousin-Poitou-CharentesFrance; 6 Hepatogastroenterology Department 26900 CHU LyonLyonAuvergne-Rhône-AlpesFrance

**Keywords:** endoscopy lower GI tract, endoscopic resection (polypectomy, ESD, EMRc, ...), colorectal cancer, quality and logistical aspects, sedation and monitoring, performance and complications

## Abstract

**Background and Aims** Colorectal endoscopic submucosal dissection (ESD) is established for large superficial neoplasia, but systemic adverse events remain underexplored. We aimed to assess the frequency, predictors, and outcomes of clinically evident acute kidney injury (AKI) after colorectal ESD.

**Methods** All consecutive colorectal ESDs performed at Limoges University Hospital (2017–2024) within the prospective French ESD Colorectal Cohort (FECCO) were retrospectively analyzed to identify AKI events and related factors.

**Results** Among 1537 patients, the frequency of clinically evident AKI was 1.3% ( *n* = 20). Compared with non-AKI patients, AKI-patients were older (74 vs. 70 years, *p* = 0.029) and more often receiving angiotensin-converting enzyme (ACE) inhibitors (40% vs. 13.6%, *p* = 0.003). AKI-patients had larger lesions (100 vs. 55 mm, *p* < 0.001), longer procedures (181 vs. 55 min, *p* < 0.001), and higher rates of perforation (35% vs. 10.4%, *p* = 0.003) and delayed bleeding (36% vs. 5.4%, *p* = 0.003). ACE-inhibitors (OR 3.96, 95% CI 1.40–9.36) and lesion diameter (OR 1.03, 95% CI 1.02–1.04) were independent predictors of AKI. By the end of follow-up, all patients recovered their baseline renal function.

**Conclusions** AKI after colorectal ESD is rare but clinically relevant. Patients receiving ACE inhibitors and with larger lesions are at higher risk. Careful periprocedural management, including adequate hydration, close monitoring during prolonged or complex procedures, and consideration of ACE inhibitor therapy, may be relevant in this setting.

## Introduction

Colorectal cancer (CRC) remains one of the most prevalent malignancies and a major cause of cancer-related mortality worldwide. 1

The increasing availability of advanced endoscopic resection techniques has transformed the management of early-stage CRC, allowing minimally invasive, organ-preserving treatment in selected patients. Endoscopic submucosal dissection (ESD) is now recognized as a potentially curative approach, enabling *en bloc* resection of large and complex lesions with accurate histologic assessment. 2

While initially developed in Asian countries, colorectal ESD has been progressively adopted in Western practice. Multicenter experiences and randomized studies have confirmed its feasibility, oncologic efficacy, and growing safety profile. 3 Technical refinements, including traction methods, have further broadened its applicability and improved outcomes. 4

Recent data from European cohorts, as well as a recent randomized controlled trial, further reinforce the efficacy, safety, and therapeutic role of colorectal ESD. 5 6 7

Despite its advantages, ESD remains a demanding procedure that may be associated with adverse events, most commonly delayed bleeding and perforation. 8 9 10 Other adverse events, however, may be less evident, particularly systemic complications not directly related to dissection itself.

In our institutional cohort, we observed cases of acute kidney injury (AKI) following colorectal ESD. Although infrequent, these events were noteworthy and prompted us to further investigate the potential association between ESD and AKI, aiming to determine whether this event could represent a procedure-related adverse outcome.

## Patients and Methods

### Study Design

This study was based on a prospective cohort including all colorectal ESD procedures performed at Limoges University Hospital (CHU Limoges) between January 2017 and December 2024 recorded in the prospective FECCO. From this prospective cohort, a retrospective analysis was conducted to assess the occurrence of AKI and its associated risk factors. This study was approved by the Institutional Review Board with a waiver of informed consent for retrospective data use.

### Inclusion and Exclusion Criteria

All colorectal ESD procedures performed from January 2017 and December 2024 were considered for inclusion. Submucosal lesions, neuroendocrine tumors, and non-neoplastic lesions were excluded from the analysis.

### Data Collection

Demographic, clinical, and procedural data were retrieved from electronic medical records and the institutional ESD database. The following variables of interest were included:

- **Patient-related factors:** age, sex, American Society of Anaesthesiologists (ASA) score, and use of anticoagulant or antiplatelet drugs were collected prospectively at the time of the procedure. Body mass index (BMI), comorbidities (arterial hypertension, type 2 diabetes mellitus, chronic kidney disease, cardiovascular disease), and chronic medications (including ACE-inhibitors, angiotensin receptor blockers (ARBs), and diuretics) were obtained retrospectively from medical records.
- **Lesion-related factors (all collected prospectively):** tumor location (rectum vs. left colon vs. right colon), size, histology, and invasion depth. Right colon lesions included those located in cecum, ascending colon, and transverse colon, while left colon lesions comprised those in the descending and sigmoid colon.
- **Procedure-related factors:** procedure duration, postprocedural bleeding, use of hemostatic interventions, occurrence of perforation, indication and timing of secondary surgery, and duration of hospital stay beyond one night were all recorded prospectively. All procedures were performed under general anesthesia. Intraoperative fluid management consisted exclusively of crystalloid solutions, administered on the basis of each patient’s clinical status during the procedure. Both the total infused crystalloid volume (mL) and the volume normalized to body weight and procedure duration (mL/kg/min) were recorded. Information on bowel preparation was retrospectively retrieved from the medical records. Intraoperative hypotension was defined according to the PeriOperative Quality Initiative (POQI) consensus statement as a mean arterial pressure (MAP) < 65 mmHg. 11
- **Laboratory data:** in patients presenting periprocedural complications or clinical symptoms, blood tests were performed; asymptomatic patients underwent clinical evaluation and were typically discharged after 24 h without biochemical control. In those diagnosed with AKI, serum creatinine values preprocedure, postprocedure (within 48–72 h from ESD) and at 6-month follow-up after the AKI event were collected retrospectively from the hospital laboratory system. Post-ESD serum creatinine was measured in patients who developed periprocedural complications or presented clinical symptoms suggesting hemodynamic instability, oliguria, or delayed recovery.

### Acute Kidney Injury

AKI was defined and staged for severity according to the Kidney Disease: Improving Global Outcomes (KDIGO) 12 criteria as reported in Table 1 .

**Table 1:** AKI stages.

Stage	Serum creatinine	Urine output
**1**	1.5–1.9 times baseline OR ≥0.3 mg/dL (≥26.5 μmol/L) increase	<0.5 mL/kg/h for 6–12 h
**2**	2.0–2.9 times baseline	<0.5 mL/kg/h for ≥12 h
**3**	3.0 times baseline OR increase in serum creatinine to ≥4.0 mg/dL (≥353.6 μmol/L) OR Initiation of renal replacement therapy OR, in patients <18 years, decrease in eGFR to <35 mL/min per 1.73 m ^2^	<0.3 ml/kg/h for ≥24 h OR Anuria for ≥12 h

### Endoscopic Submucosal Dissection

In our unit, during the study period, we performed colorectal ESD, applying a Japanese approach, for all large (>25 mm) laterally spreading tumors (LSTs). Single-channel high-resolution endoscopes were used for ESD, with patients under general anesthesia. After detailed optical diagnosis confirming the resectability of the lesion, a complete or partial incision was performed using an endoscopic knife. The strategy for submucosal dissection was at the discretion of the operators, but our team has developed and frequently uses a double-clip traction strategy with clips and rubber bands. VIO 3 electrosurgical units (Erbe Elektromedizin, Tübingen, Germany) was used. EndoCUT I was used for mucosal incision, and swiftCOAG, preciseSECT, or endoCUT I was used for submucosal dissection. At the end of the procedure, visible vessels were systematically prophylactically coagulated using monopolar coagulation forceps. We did not include adrenaline in the injection solution and did not routinely close mucosal defects after ESD. Owing to geographical and financial constraints, all patients remained in hospital for 1 night after ESD. Only patients who experienced adverse events had prolonged hospitalization of more than 1 night after ESD.

### Study Outcomes

The primary endpoint was the frequency of clinically evident AKI after colorectal ESD. The secondary endpoint was the identification of independent prognostic factors associated with the development of AKI, including patient-, lesion-, and procedure-related variables.

### Statistical Analysis

Continuous variables were expressed as medians with interquartile ranges (25th–75th percentiles), while categorical variables were presented as absolute numbers and relative frequencies (%). Patients were divided into two groups according to the occurrence or not of AKI after colorectal ESD. The normality of continuous variables was tested using the Shapiro–Wilk test, and homogeneity of variances was assessed with Levene’s test. Depending on data distribution, continuous variables were compared using the unpaired Student’s *t* test, or the Mann–Whitney *U* test. Categorical variables were compared using the chi-squared test or Fisher’s exact test, as appropriate. Correlation between variables was assessed using two-tailed Spearman’s rank correlation, and 95% confidence intervals were calculated for all correlation coefficients.

Univariable and multivariable analyses were performed to identify factors associated with AKI development. To account for potential bias due to small events size, we performed Firth’s penalized logistic regression, which applies a penalization based on Jeffreys’ prior to the likelihood function, providing finite and less biased estimates of odds ratios and confidence intervals.

The alpha risk was set at 5%, and all statistical tests were two-sided. Statistical significance was considered if *p* value < 0.05.

Statistical analyses were performed using EasyMedStat (version 3.44; www.easymedstat.com ) and SPSS (version 29.0).

Missing data for baseline variables were handled using complete-case analysis. Given the low proportion of missing values (<10%), no imputation was performed, as this approach is generally considered acceptable in this context. The impact on the overall results is therefore expected to be minimal.

## Results

### Baseline Characteristics of Patients and Lesions ( Table 2 )

**Table 2:** Patient and lesion characteristics.

Characteristics	Number of patients with available data (missing patients %)	Value ( *n* = 1537) *n* (%), median (IQR)
Age, years	1536 (0.06)	70 (63–75)
Sex M F	1537 (0)	884 (57.5) 653 (42.5)
ASA score 1 2 3 4	1534 (0.2)	212 (13.8) 768 (50.1) 530 (34.6) 24 (1.6)
BMI, Kg/m ^2^	1479 (3.77)	25.71 (23.06–28.73)
Obesity, yes	1477 (3.9)	275 (18.6)
Diabetes, yes	1449 (5.73)	200 (13.8)
High blood pressure, yes	1448 (5.79)	647 (44.7)
CVD, yes	1447 (5.86)	221 (15.3)
CKD, yes G2 G3a G3b G4 G5	1450 (5.66)	58 (4) 17 (1.1) 20 (1.3) 16 (1) 3 (0.2) 2 (0.1)
Drugs ACE inhibitor Angiotensin II receptor blockers Diuretic	1450 (5.66) 1450 (5.66) 1449 (5.73)	202 (13.9) 262 (18.1) 224 (15.5)
Anticoagulant, yes	1537 (0)	159 (10.3)
Anti-platelets, yes	1537 (0)	263 (17.1)
Localization Rectum Left colon Right colon	1529 (0.52)	528 (34.5) 342 (22.4) 659 (43.1)
Type of lesion GH LST GM LST Protruding lesion Flat NG LST Pseudo-depressed NG LST	1505 (2.08)	452 (30) 514 (34.2) 212 (14.1) 231 (15.3) 96 (6.4)
JNET classification JNET I JNET IIa JNET IIb JNET III	1499 (2.47)	18 (1.2) 983 (65.6) 469 (31.3) 29 (1.9)
CONECCT classification Is IIa IIc IIc+ III	1507 (1.95)	32 (2.1) 453 (30.1) 986 (65.4) 8 (0.5) 28.0 (1.9)
Largest diameter, mm	1507 (1.95)	55 (45–70)
Procedure duration, min	1500 (2.41)	56 (45–70)
Speed of dissection, mm ^2^ /min	1474 (4.1)	37.5 (24.7–54.0)
Fibrosis F0 F1 F2	1517 (0.13)	481 (31.7) 571 (37.6) 465 (30.7)
Perforation, yes	1537 (0)	164 (10.7)
Delayed bleeding, yes	1533 (0.26)	87 (5.7)
Need of prolonged hospitalization (>1 night), yes	1537 (0)	204 (13.3)
*En bloc* resection, yes	1537 (0)	1479 (96.2)
R0, yes	1525 (0.78)	1359 (89.1)
Lesion histology Ssl LGD adenoma HGD adenoma/intramucosal cancer SM<1000 mm cancer SM>1000 mm cancer T2 cancer	1523 (0.91)	51 (3.3) 792 (52) 517 (33.9) 68 (4.5) 86 (5.6) 9 (0.6)
Secondary surgery, yes	1537 (0)	88 (5.7)
Reason of secondary surgery Endoscopic adverse events Pathological analysis Failure	1537 (0)	12 (0.8) 51 (3.3) 18 (1.2)
Post-procedural AKI	1537 (0)	20 (1.3)

ASA, American Society of Anaesthesiologists; BMI, body mass index; CVD, cardiovascular disease; CKD, chronic kidney disease; ACE, angiotensin-converting enzyme; GH LST, granular homogeneous lateral spreading tumor; GM LST, granular mixed lateral spreading tumor; NG LST, non granular lateral spreading tumor; JNET, Japan NBI Expert Team III; CONECCT, Colorectal Neoplasia Endoscopic Classification to Choose Treatment; LGD, low grade dysplasia; HGD, high grade dysplasia; SM submucosal; Ssl, sessile serrated lesion; AKI, acute kidney injury.

After applying exclusion criteria, 1537 patients were included in the final analysis ( Fig. 1 ). The majority were male (57.5%), with a median age of 70 years (IQR 63–75). Most patients were classified as ASA II (50.1%) or ASA III (34.6%). The most frequent comorbidity was hypertension (44.7%), while obesity, diabetes mellitus, and cardiovascular disease were each present in fewer than 20% of patients. Chronic kidney disease (CKD) was documented in 4% of cases. Regarding chronic medications, 13.9% were receiving ACE inhibitors, 18.1% ARBs, and 15.5% diuretics. Most lesions were located in the right colon (43.1%) and were classified as granular-mixed LSTs (34.2%). The median largest lesion diameter was 55 mm (IQR 45–70).

**Fig. 1:**
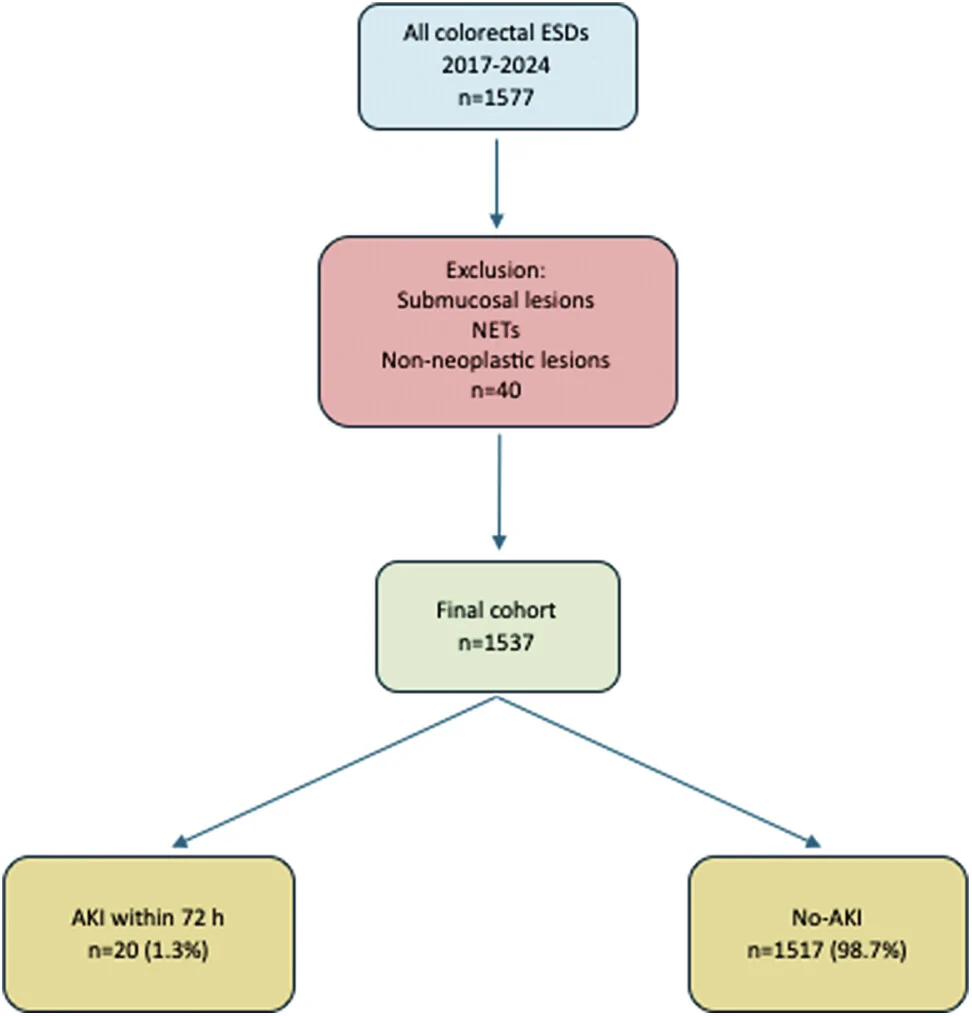
Study flow-chart.

### ESD Characteristics ( Table 2 )

The median procedure duration was 56 min (IQR 45–70). En bloc resection was achieved in 1479 patients (96.2%), with R0 resection in 1359 (89.1%). The median dissection speed was 37.5 mm²/min (IQR 24.7–54). Perforation and postprocedural bleeding occurred in 10.7% and 5.7% of cases, respectively. The overall rate of surgery for endoscopic complications was 0.8%. Median length of hospital stay was 1 day (IQR 1–1), and only 204 patients (13.3%) required prolonged hospitalization (>1 night).

### Risk Factors and Predictors of Acute Kidney Injury following ESD

Within the first 72 h after ESD, 20 patients (1.3%) developed clinically evident AKI. These patients were significantly older than those without AKI (median 74 vs. 70 years, *p* = 0.029) and more frequently treated with ACE inhibitors (40% vs. 13.6%, *p* = 0.003; OR 4.25, 95% CI 1.71–10.5). In contrast, sex; ASA score; comorbidities (including obesity, diabetes mellitus, hypertension, and cardiovascular disease); and use of anticoagulants, antiplatelets, or diuretics were not significantly associated with AKI ( Tables 3 and 4 ).

**Table 3:** Comparison between non-AKI vs. AKI patients.

Variables	Number of patients with available data (missing patients%)	Non-AKI ( *n* = 1517) *n* (%), median (IQR)	AKI ( *n* = 20) *n* (%), median (IQR)	*p* Value	OR (95%CI)
Age, years	1536 (0.06)	70 (63–75)	74 (64.8–81.8)	**0.029**	
Sex, M	1537 (0)	874 (57.6)	10 (50.0)	0.504	1.359 (0.562 – 3.28)
ASA score 1 2 3 4	1534 (0.2)	210 (13.9) 761 (50.3) 520 (34.4) 23 (1.5)	2 (10) 7 (35) 10 (50) 1 (5)	0.171	
BMI, Kg/m ^2^	1479 (3.77)	25.7 (23.1–28.7)	24.9 (22.6–27.1)	0.292	
Obesity, yes	1479 (3.77)	272 (18.6)	3 (15)	0.474	0.769 (0.224–2.646)
Diabetes, yes	1449 (5.73)	197 (13.8)	3 (15)	0.749	1.104 (0.32–3.8)
High. Blood pressure, yes	1448 (5.79)	636 (44.5)	11 (55)	0.373	1.522 (0.627–3.7)
CVD, yes	1447 (5.86)	217 (15.2)	4 (20)	0.531	1.394 (0.462–4.21)
CKD, yes	1450 (5.66)	58 (4.1)	0 (0)	>0.999	
ACE inhibitor, yes	1450 (5.66)	194 (13.6)	8 (40)	**0.003**	4.25 (1.71–10.5)
Angiotensin II receptor blockers, yes	1450 (5.66)	261 (18.3)	1 (5)	0.152	0.236 (0.031–1.77)
Diuretic, yes	1449 (5.73)	222 (15.5)	2 (10)	0.756	0.604 (0.139–2.62)
Anticoagulant, yes	1537 (0)	157 (10.4)	2 (10)	>0.999	0.962 (0.221–4.19)
Anti-platelet, yes	1537 (0)	258 (17)	5 (25)	0.366	1.626 (0.586–4.52)
Localization Right colon Left colon Rectum	1529 (0.52)	656 (43.5) 339 (22.5) 514 (34.1)	3 (15) 3 (15) 14 (70)	**0.003**	
Localization, rectum	1529 (0.52)	514 (34.1)	14 (70)	**<0.001**	**4.517 (1.726 –11.824)**
Type of lesion GH LST GM LST Protruding lesion Flat NG LST Pseudo-depressed NG LST	1505 (2.08)	449 (30.2) 499 (33.6) 211 (14.2) 231 (15.6) 95 (6.4)	3 (15) 15 (75) 1 (5) 0 (0) 1 (5)	**0.005**	
Type of lesion GM LST	1505 (2.08)	499 (33.6)	15 (75)	**<0.001**	**5.928 (2.142 –16.404)**
Largest diameter, mm	1507 (1.95)	55 (45–70)	100 (70–150)	**<0.001**	
Procedure duration, min	1500 (2.41)	55 (36–90)	181.0 (103–267.5)	**<0.001**	
Speed of dissection, mm ^2^ /min	1474 (4.1)	37.5 (24.5–53.8)	45.8 (36.4–61.1)	0.186	
Injection solution for dissection Cellulose-based & hypertonic Viscous gel Succinylated gelatine Glycerol Water and poloxamers NaCl Hydroxyethyl starch and NaCl	1519 (1.17)	8 (0.5) 6 (0.4) 36 (2.4) 1428 (95.3) 2 (0.1) 15 (1) 4 (0.3)	0 (0) 0 (0) 0 (0) 20 (100) 0 (0) 0 (0) 0 (0)	>0.999	
Fibrosis F0 F1 F2	1517 (1.3)	478 (31.9) 565 (37.7) 455 (30.4)	3 (15.8) 6 (31.6) 10 (52.6)	0.093	
Perforation, yes	1537 (0)	157 (10.4)	7 (35)	**0.003**	**4.664 (1.83–11.86)**
Delayed bleeding, yes	1533 (0.26)	82 (5.4)	5 (26.3)	**0.003**	**6.237 (2.19–17.74)**
Hypotension during procedure, yes	1373 (10.67)	518 (38.3)	11 (55)	0.164	1.970 (0.811–4.79)
Total infused volume, mL	1432 (6.83)	1000 (500–1000)	1500 (1000–1500)	**<0.001**	
Total infused volume/bodyweight/duration mL/kg/min	1402 (8.78)	0.19 (0.12–0.32)	0.11 (0.07–0.15)	**<0.001**	
*En bloc* resection, yes	1537 (0)	1462 (96.4)	17 (85)	**0.037**	**0.213 (0.061–0.749)**
R0, yes	1525 (0.78)	1349 (89.5)	10 (55.6)	**<0.001**	0.146 (0.057–0.376)
Lesion histology Ssl LGD adenoma HGD adenoma/intramucosal cancer SM < 1000 mm cancer SM > 1000 mm cancer Cancer T2	1523 (0.91)	50 (3.3) 785 (52.2) 509 (33.8) 67 (4.5) 84 (5.6) 19 (0.6)	1 (5.3) 7 (36.8) 8 (42.1) 1 (5.3) 2 (10.5) 0 (0)	0.415	
Length of hospitalization, days	1536 (0.06)	1 (1–1)	9.0 (5–15.3)	**<0.001**	
Bowel preparation Sodium picosulfate Sodium sulfate Polyethylene glycol Macrogol Others	880 (42.7)	12 (1.4) 73 (8.5) 461 (53.4) 312 (36.2) 5 (0.6)	0 (0) 0 (0) 12 (70.6) 4 (23.53) 1 (5.9)	0.12	

OR, odds ratio; CI, confidence interval; ASA, American Society of Anaesthesiologists; BMI, Body Mass Index; CVD, Cardiovascular disease; CKD, Chronic Kidney Disease; ACE, Angiotensin-converting enzyme; GH LST, granular-homogeneous laterally spreading tumor; GM LST, granular-mixed laterally spreading tumor; NG LST, non-granular laterally spreading tumor; JNET, Japan NBI Expert Team classification; CONECCT, Colorectal Neoplasia Endoscopic Classification to Choose Treatment; R0, histologically complete resection (negative margins); LGD, low-grade dysplasia; HGD, high-grade dysplasia; SM, submucosal invasion; Ssl, sessile serrated lesion; MAP, mean arterial pressure; AKI, acute kidney injury.

**Table 4:** Univariable and multivariable analysis for AKI development in patients undergoing ESD.

Variable	Univariable analysis			Multivariable analysis		
	OR	95% CI	*p*	OR	95% CI	*p*
Largest diameter	1.04	1.03 – 1.05	<0.001	1.03	1.02−1.04	<0.001
Localization of the lesion (rectum vs. colon)	4.32	1.72–10.36	0.003	2.52	0.90−7.89	0.080
Ace-inhibitor treatment	4.31	1.77–11.78	0.001	3.96	1.40−9.36	0.011

OR, odds ratio; CI, confidence interval; ACE, angiotensin-converting enzyme. Lesion largest diameter is expressed in millimetres (mm); procedure duration in minutes (min).

Lesion characteristics also differed: granular-mixed LSTs and rectal lesions were associated with a higher risk of AKI (OR 5.93, 95% CI 2.14–16.40, and OR 4.52, 95% CI 1.73–11.82, respectively). Procedural factors were strongly associated with AKI. Patients who developed AKI had longer procedures [181 min (IQR 103–268) vs. 55 min (IQR 36–90), *p* < 0.001], larger lesions [100 mm (IQR 70–150) vs. 55 mm (IQR 45–70), *p* < 0.001], and higher rates of perforation (35% vs. 10.4%, *p* = 0.003) and postprocedural bleeding (36.3% vs. 5.4%, *p* = 0.003). Notably, when total infused volume was normalized for body weight and procedure duration (mL/kg/min), patients with AKI received significantly less fluid than those without AKI [0.11 (IQR 0.07–0.15) vs. 0.19 (IQR 0.12–0.32), *p* < 0.001]. The incidence of intraprocedural hypotension did not differ significantly between groups.

In multivariable analysis, lesion diameter and preprocedural ACE inhibitor therapy remained independently associated with AKI, with ACE inhibitor therapy being the strongest predictor (OR 3.96, 95% CI 1.40–9.36). Procedure duration was excluded due to collinearity with lesion size.

### Outcomes of Patients with AKI ( Table 5 )

**Table 5:** AKI patient’s characteristic.

Patients	Age	ASA score	BMI	Diabetes	CVD	ACE inhibitors	Lesion localization	Lesion size (Largest diameter, mm)	Hypotension during procedure	Delayed bleeding	Use of vasopressor	Cr pre-procedure, μmol/L	Cr post- rocedure, μmol/L	Reduced urinary output	AKI stage	Dialysis	Kidney biopsy	Histology	Length of hospitalization, days	Cr at 6 months from discharge	DCr	DCr2
**1**	89	3	30.4	No	No	No	Rectum	195	No	Yes	No	82	880	Yes	3	Yes	Yes	Acute tubular necrosis with chronic fibrous interstitial lesion	25	72	808	−10
**2**	87	2	24.2	No	No	No	Left Colon	65	Yes	No	Yes	69	280	Yes	3	No	No		16	78	202	9
**3**	89	2	32	No	No	No	Rectum	130	Yes	No	Yes	99	188	Yes	1	No	No		5	100	88	1
**4**	88	3	23.6	No	No	Yes	Rectum	60	Yes	No	Yes	78	233	Yes	2	No	No		4	80	153	2
**5**	81	3	26.6	No	No	Yes	Right Colon	65	Yes	No	Yes	70	139	No	1	No	No		5	73	66	3
**6**	78	3	29	No	No	Yes	Rectum	97	Yes	No	Yes	107	212	Yes	2	No	No		14	110	102	3
**7**	81	3	23.9	No	No	Yes	Rectum	150	No	No	No	54	244	Yes	3	No	No		6	62	182	8
**8**	76	1	19.6	No	No	Yes	Rectum	95	Yes	No	Yes	64	300	Yes	3	No	No		7	65	235	1
9	69	3	25.7	Yes	Yes	No	Right Colon	60	Yes	Yes	Yes	142	182	No	1	No	No		5	137	40	-5
**10**	73	4	18.7	No	No	No	Rectum	160	Yes	Yes	Yes	35	100	Yes	2	No	No		23	49	51	14
**11**	75	3	26.8	Yes	Yes	Yes	Rectum	190	No	No	No	107	1090	Yes	3	No	No		3	107	983	0
**12**	73	2	25.5	Yes	No	Yes	Rectum	150	No	No	No	82	974	Yes	3	No	No		17	85	889	3
**13**	72	3	33.4	No	Yes	Yes	Right Colon	70	No	Yes	No	89	132	No	1	No	No		4	87	35	−2
**14**	65	2	23.5	No	No	No	Rectum	75	Yes	No	No	70	300	Yes	3	No	No		8	72	228	2
**15**	64	2	27.7	No	Yes	No	Rectum	75	No	No	No	77	930	Yes	3	No	Yes	Moderate acute tubulointerstitial injury	21	96	834	19
**16**	63	3	19.6	No	No	No	Left Colon	130	No	Yes	No	69	640	Yes	3	No	Yes	Acute tubular necrosis without microvacuolization; occasional microcalcifications with calcium component	13	60	580	−9
**17**	60	2	23.5	No	No	No	Rectum	100	Yes	No	Yes	92	122	Yes	1	No	No		2	90	32	−2
**18**	62	2	27	No	No	No	Rectum	100	No	No	No	60	670	Yes	3	No	No		10	66	604	6
**19**	64	3	16.2	No	No	No	Left Colon	65	Yes	No	No	81	600	Yes	3	No	No		10	87	513	6
**20**	84	1	19.8	No	No	No	Rectum	185	No	No	No	100	922	Yes	3	No	No		15	95	827	-5

ASA, American Society of Anaesthesiologists; BMI, Body Mass Index; ACE, Angiotensin-converting enzyme; Reduced urinary output: <0.5 mL/kg/h during >6 h. DCr: difference between 6 months-creatinine and post-procedural creatinine. DCr2: difference between 6 months-creatinine and pre-procedural creatinine. Hypotension during procedure: MAP (mean arterial pressure) <65 mmHg in any moment, AKI, acute kidney injury; CVD, cardiovascular disease: documented history of myocardial infarction, angina or coronary revascularization, stroke or transient ischemic attack, peripheral arterial disease, heart failure, valvular heart disease, or clinically significant arrhythmias.

Among the 20 patients with clinically evident AKI, median pre-ESD creatinine was 79 μmol/L (IQR 69–93), which increased to 290 μmol/L (IQR 186–722) post-procedure. Vasopressors were required in 9 out of 11 patients with AKI who experienced intra-procedural hypotension. All but two patients developed oliguria (<0.5 mL/kg/h for >6 h), and one patient (5.0%) required two sessions of hemodialysis. Renal biopsy was performed in three patients, all demonstrating moderate-to-severe acute tubular necrosis. Median hospital stay was 9 days (IQR 5–15.3), significantly longer than in patients without AKI ( *p* < 0.001). However, all patients recovered baseline renal function, with follow-up creatinine values ranging from –10 to +19 μmol/L compared with pre-ESD levels.

## Discussion

To our knowledge, this is the first study to systematically evaluate the frequency and risk factors of clinically evident AKI following colorectal ESD. We observed a very low frequency (1.3%), markedly lower than that reported after colorectal surgery, where AKI rates of approximately 13–20% have been described in large national and ERAS cohorts. 13 14 This difference likely reflects the minimally invasive nature of ESD, which avoids the systemic stress, major fluid shifts, and inflammatory activation associated with open or laparoscopic surgery. Nevertheless, our findings highlight specific risk factors: older age, larger lesions, longer procedures, and rectal localization were all associated with increased risk. Importantly, preprocedural ACE inhibitor therapy emerged as the strongest independent predictor, consistent with surgical series in which renin–angiotensin system blockade impairs renal autoregulation and predisposes to periprocedural ischemic injury. 15 16

In our unit, anesthesia during ESD is maintained with inhaled halogenated agents such as sevoflurane. Beyond their hypnotic properties, these agents induce peripheral vasodilation and decrease systemic vascular resistance. 17 When combined with preprocedural ACE inhibitor therapy, this effect may further reduce effective circulating blood volume and compromise renal perfusion. This synergistic hemodynamic effect provides a plausible explanation for the increased susceptibility to AKI observed in patients receiving ACE inhibitors, particularly during longer or more complex procedures.

As expected, we observed a significant correlation between procedure duration and lesion size ( *R* = 0.649, 95% CI 0.619–0.678, *p* < 0.001) ( Fig. 2 ). Larger lesions are also consistently associated with lower en bloc and R0 resection rates, 18 higher risk of delayed bleeding, 10 and greater risk of perforation. 19 Notably, large lesions are most often granular-mixed LSTs. 20 Collectively, these factors translate into prolonged procedures, increased tissue manipulation, and potentially greater hemodynamic instability, all of which can predispose to AKI ( Fig. 3 ).

**Fig. 2:**
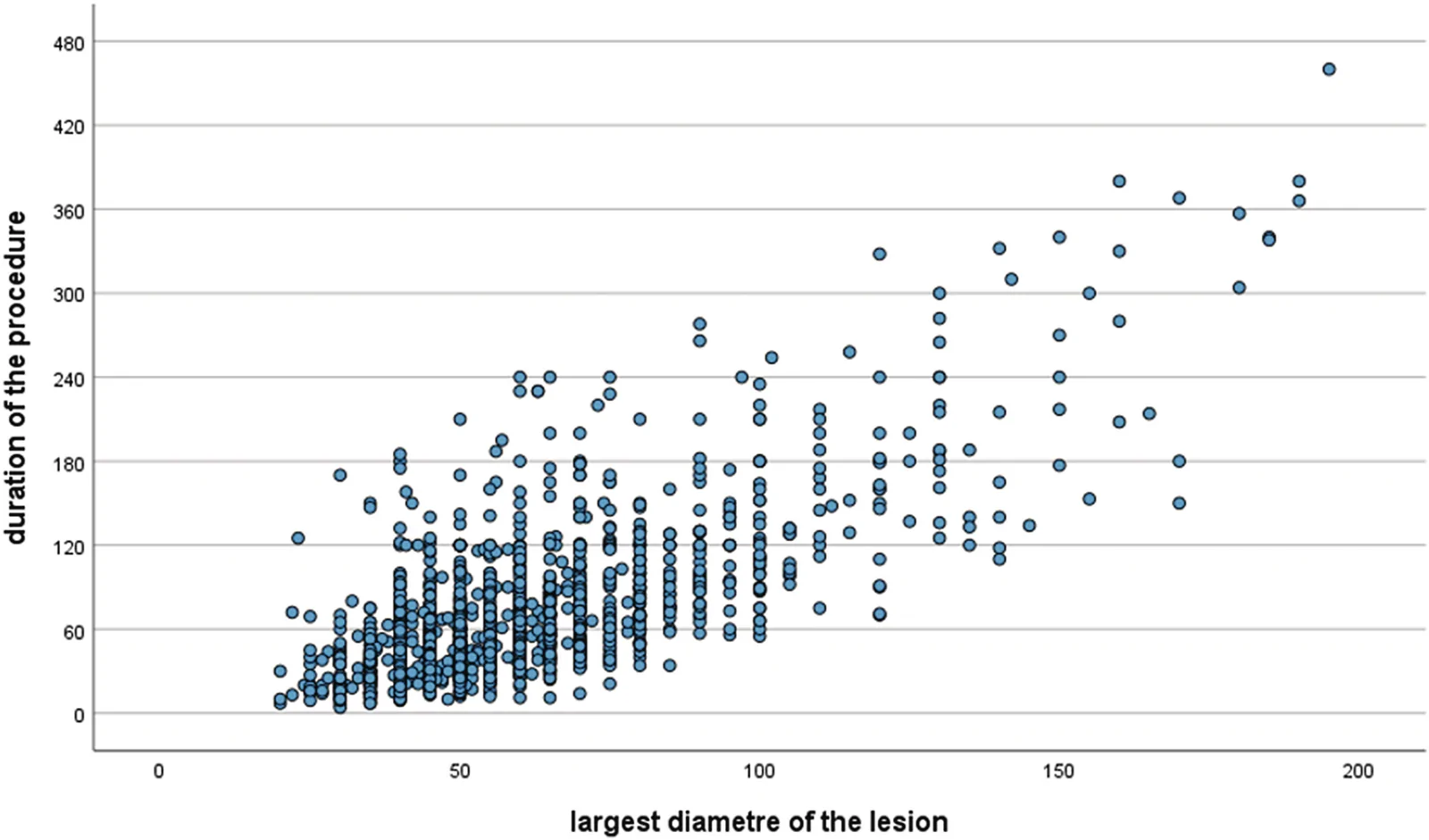
Spearman correlation between procedure duration and lesion largest diameter.

**Fig. 3:**
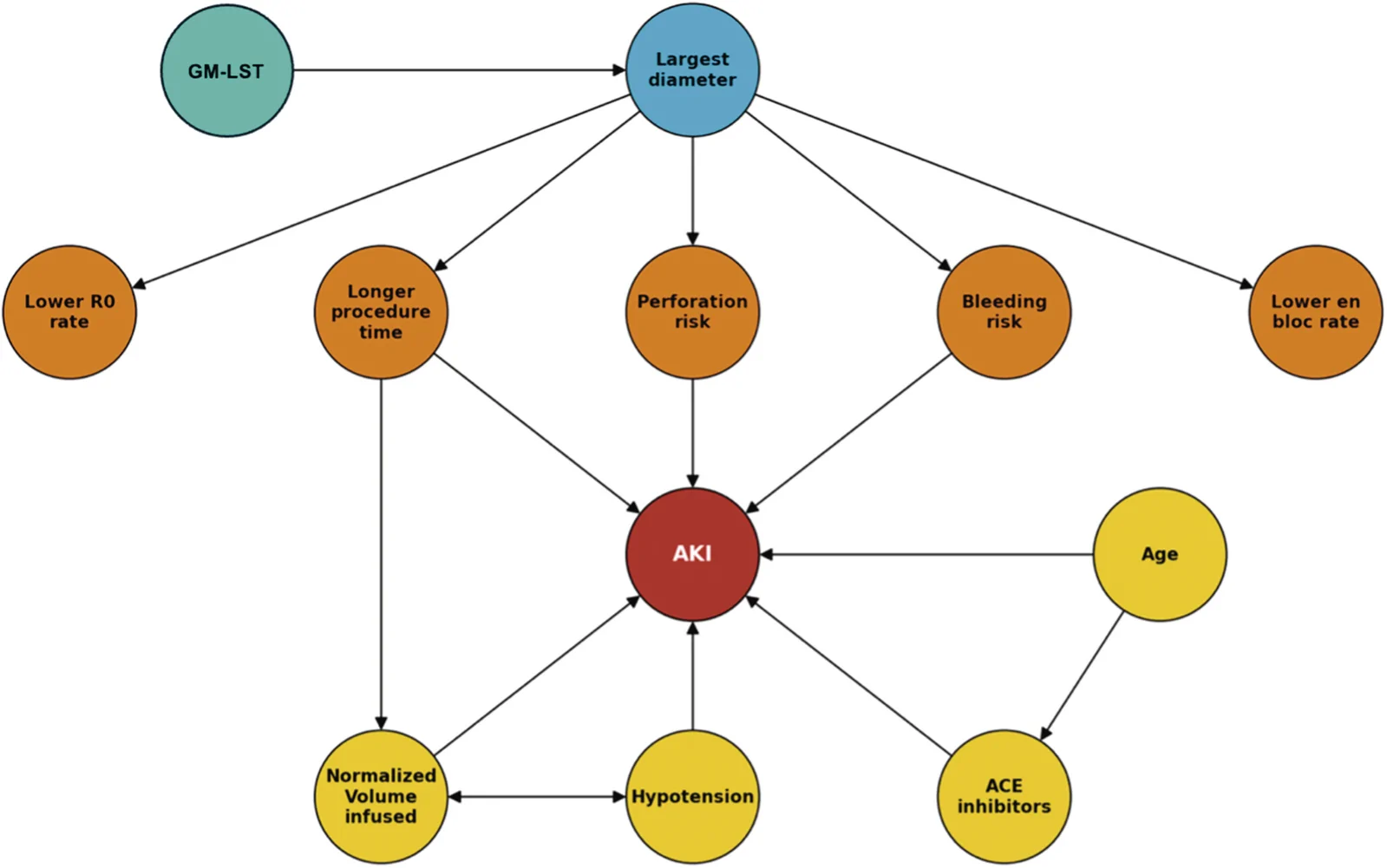
Direct Acyclic Graphic for risk factors correlated to AKI development after ESD.

Interestingly, although patients with clinically evident AKI received a higher absolute volume of fluids, their weight- and time-adjusted infusion rate was significantly lower than that of non-AKI patients. This apparent paradox suggests that prolonged procedures, often complicated by bleeding or perforation, required larger rescue infusions but still resulted in a relatively restrictive fluid strategy over time. This finding echoes recent concerns in colorectal surgery, where overly restrictive fluid management under ERAS protocols has been associated with an increased risk of AKI. 21 22 This mechanism, potentially compounded by dehydration related to previous bowel preparation, may be further exacerbated in patients receiving renin–angiotensin system inhibitors, in whom renal perfusion is more dependent on adequate intravascular volume. Optimizing periprocedural hydration and closely monitoring urinary output may therefore be crucial in preventing renal injury. All patients in our cohort received submucosal injection with glycerol; while hyperosmolar solutions could theoretically contribute to renal stress, this could not be evaluated as a risk factor given the universal exposure.

The association between clinically evident AKI and rectal lesions, although not fully independent, remains clinically plausible. In our cohort, rectal lesions were almost exclusively managed using CO₂ insufflation without the underwater technique. Prolonged insufflation during these procedures may lead to increased intra-abdominal pressure, potentially resulting in elevated renal venous pressure and reduced renal blood flow. However, intra-abdominal pressure was not directly measured in this study, and this mechanism should therefore be interpreted with caution, although similar effects of intra-abdominal hypertension on renal perfusion have been described in surgical and critical care settings. 23 In our series, renal biopsies confirmed acute tubular necrosis in patients with AKI, providing histologic support for this mechanism. However, this association should be interpreted with caution, as rectal lesions were also larger and associated with longer procedure time, both of which were more frequent among patients who developed AKI in our cohort. Although delayed bleeding was more common in AKI patients, it usually occurred after AKI onset and is therefore unlikely to represent a primary causal factor in most cases.

Although the frequency of clinically evident AKI was low, its clinical impact was significant, with prolonged hospitalization and one case requiring dialysis. Beyond the individual burden, extended hospital stays also increase healthcare costs, underlining the broader implications of this complication. Encouragingly, all patients recovered their baseline renal function.

The strengths of this study include prospective data collection and a large sample size, which enabled the identification of a rare but clinically relevant complication.

Several limitations must also be acknowledged. The relatively small number of clinically evident AKI events limited the number of variables that could be included in multivariable analysis. Residual confounding cannot be excluded despite the use of a prespecified adjustment set and penalized regression. Additionally, detailed information regarding the duration or depth of intraprocedural hypotension was not systematically recorded, limiting further analysis of its potential contribution to AKI. Because postoperative serum creatinine measurements were not systematically performed in all patients, mild or asymptomatic AKI cases may not have been captured. Accordingly, our findings likely reflect clinically evident AKI events rather than the full spectrum of renal function changes after colorectal ESD. The single-center design may also limit generalizability. Finally, the near-universal use of glycerol precluded formal dose–response testing. Limited data exist regarding the potential renal toxicity of high-volume glycerol injection. As a precaution, for large rectal lesions, we suggest that normal saline may be preferable to glycerol, given the substantial injection volumes typically required.

In conclusion, colorectal ESD is a safe and effective procedure, with a very low frequency of clinically evident AKI. Nonetheless, patient- and procedure-related factors, particularly ACE inhibitor therapy and lesion size, should be taken into account during risk assessment and periprocedural management. Careful hydration, close monitoring, and temporary discontinuation of ACE inhibitors might be considered on an individual basis in patients undergoing lengthy or complex procedures, although these strategies require further validation in prospective and interventional studies.
